# Nuclear envelope rupture and resealing: mechanisms, consequences, and disease implications

**DOI:** 10.1042/BST20250351

**Published:** 2026-09-24

**Authors:** Atsuki En, Kohta Ikegami

**Affiliations:** 1 Division of Molecular Cardiovascular Biology, Cincinnati Children’s Hospital Medical Center, Cincinnati, Ohio, U.S.A.; 2 Heart Institute, Cincinnati Children’s Hospital Medical Center, Cincinnati, Ohio, U.S.A.; 3 Department of Pediatrics, University of Cincinnati College of Medicine, Cincinnati, Ohio, U.S.A.

**Keywords:** chromosomes, DNA damage, ESCRT-III, lipids, mechanotransduction, nuclear envelopes

## Abstract

The nuclear envelope (NE) establishes an essential physical separation between the nucleoplasm and cytoplasm in eukaryotic cells. Its structural integrity ensures selective molecular exchange through nuclear pores, a fundamental feature of eukaryotic cells. For this reason, it has long been assumed that the NE remains intact. However, recent studies revealed localized rupture of the NE in diverse physiological and pathological contexts. These ruptures can be resealed by endogenous mechanisms, suggesting that rupture–resealing dynamics contribute to homeostasis and are altered in disease. In the present review, we summarize physiological and pathological contexts in which NE rupture has been observed. We evaluate major hypotheses regarding its causes and consequences and discuss current understanding of the mechanisms and regulation of NE resealing. We conclude by highlighting key knowledge gaps in the pathophysiological roles of NE rupture and resealing.

## Introduction

The nuclear envelope (NE) segregates DNA replication and transcription from translation, providing an essential regulatory layer of the central dogma in eukaryotic cells. For many years, it was believed that the NE loses its integrity only when cells divide or die. Surprisingly, however, recent research has revealed that the NE can undergo localized rupture outside these contexts without causing immediate cell death. Such rupture has been reported in a variety of pathological settings, including degenerative diseases and cancer, as well as under certain physiological conditions. In the present mini-review, we discuss the causes and consequences of NE rupture and summarize emerging insights into the roles of NE rupture and subsequent resealing in development, tissue homeostasis, and disease. We conclude by highlighting key unanswered questions, with particular emphasis on the significance of NE rupture in human disease.

## Causes of nuclear envelope rupture

The major known contributor to NE rupture is an imbalance between mechanical forces applied to the nucleus and the mechanical resistance of the NE. We discuss below how this imbalance arises in diverse cellular environments (Figure 1).

**Figure 1:**
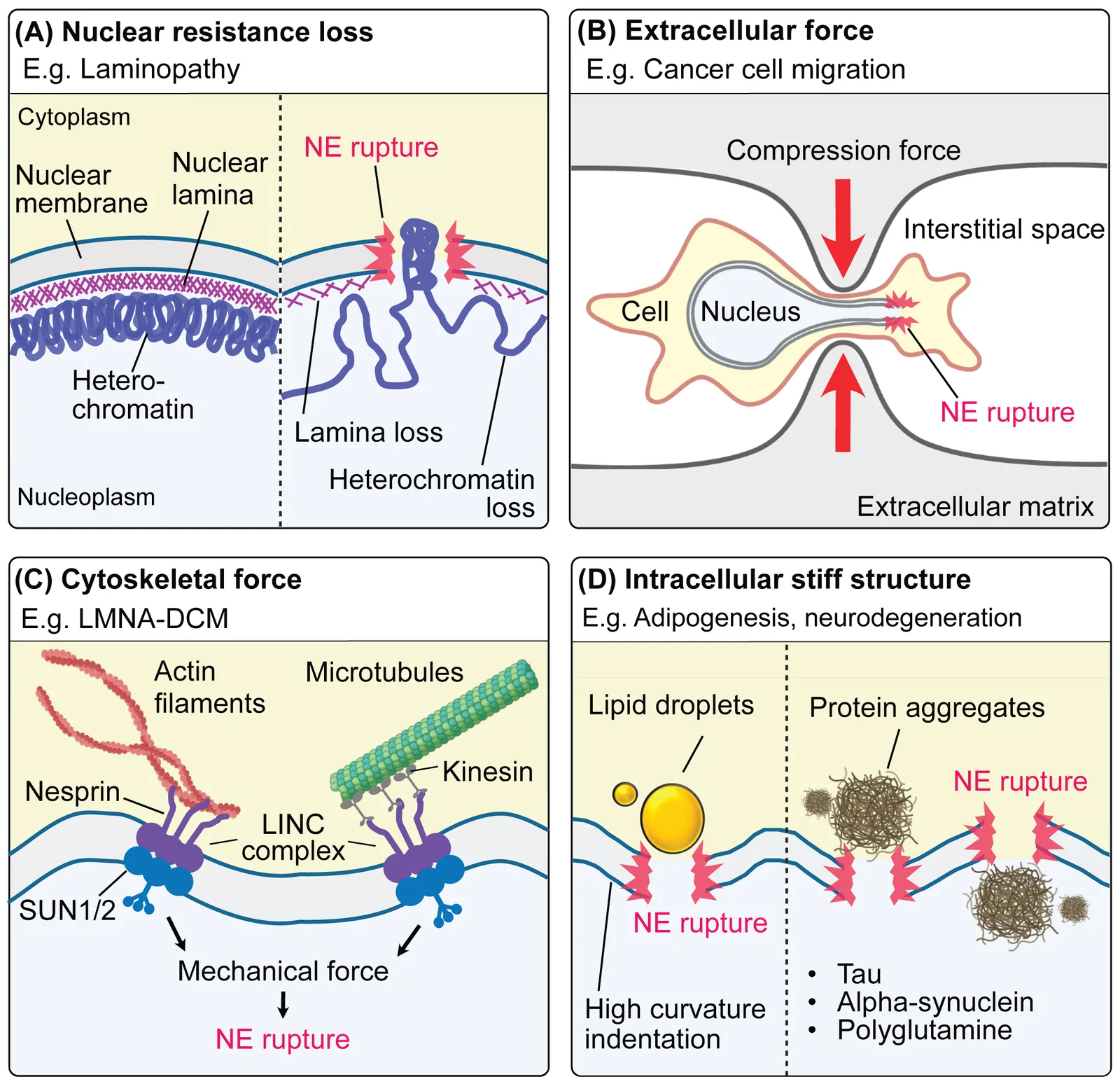
Contributors of NE rupture (**A**) Mutations in or dysfunction of nuclear lamins compromise nuclear lamina integrity and disrupt heterochromatin organization, promoting NE rupture. (**B**) Migration through constricted spaces generates compressive forces on the nucleus, leading to NE rupture. (**C**) Actin and microtubule networks exert mechanical forces on the nucleus through the linker of nucleoskeleton and cytoskeleton (LINC) complex (Nesprins and SUN1/2), contributing to NE rupture. (**D**) Local NE deformation induced by small lipid droplets (left) or protein aggregates (right) can cause NE rupture.

### Nuclear lamina and nuclear membrane alterations

Reduction or dysfunction of nuclear lamin proteins is one of the best-described contributors to NE rupture (Figure 1A). Nuclear lamins are components of the nuclear lamina, a protein meshwork composed of lamin polymers beneath the inner nuclear membrane [1,2]. The nuclear lamina provides structural support to the NE and tethers heterochromatin to the nuclear periphery. In human cells, nuclear lamins include Lamin A/C (long splice isoform Lamin A and short splice isoform Lamin C, both encoded by *LMNA*), Lamin B1 (encoded by *LMNB1*), and Lamin B2 (encoded by *LMNB2*) [1–3]. More than 400 distinct missense, nonsense, or truncating mutations in these lamin genes have been reported to cause a spectrum of degenerative diseases collectively called laminopathies [4–7]. NE rupture has been reported in cardiomyocytes [8–14], skeletal muscle cells [15,16], smooth muscle cells (SMCs) [17], macrophages [18], and fibroblasts [19–22] from laminopathy patients, animal models, or cellular models carrying Lamin A/C mutations. NE rupture resulting from Lamin B1 or B2 reduction has also been observed in neurons [23,24] and fibroblasts [24,25] in cellular and animal models. NE rupture can occur at remarkably high frequencies, affecting up to 50% of Lamin A/C-depleted cardiomyocytes [13] and 60% of Lamin B1-depleted cultured neurons [23].

There are two proposed, non-mutually exclusive mechanisms by which nuclear lamin reduction or mutations cause NE rupture (Figure 1A). The first mechanism is weakening of the nuclear lamina meshwork. The nuclear lamina exhibits silk-like mechanical properties that enable it to resist mechanical stress [26]. Therefore, its reduction or dysfunction would decrease the mechanical resistance of the NE. The second mechanism is altered organization of nuclear lamina-associated heterochromatin. Heterochromatin contributes substantially to nuclear stiffness [27–29]. Supporting this mechanism, chromatin decompaction increases susceptibility to NE rupture in various cell types, independently of changes in nuclear lamins [28,30,31]. Thus, both the nuclear lamina meshwork and lamina-associated heterochromatin likely contribute to the overall mechanical resistance conferred by nuclear lamins. Interestingly, evidence suggests that Lamin A/C provides greater mechanical resistance than Lamin B1 or B2 [32]. Because nuclear lamin expression varies across cell types and tissues [33], with Lamin A/C predominating in differentiated cells [34,35], susceptibility to NE rupture likely differs among cell types.

In addition to defects in the nuclear lamina, emerging evidence suggests that nuclear membrane lipid composition can influence susceptibility to NE rupture. One line of evidence comes from the investigation of CTDNEP1, an NE-localized phosphatase that activates Lipin1, which catalyzes the synthesis of diacylglycerol from phosphatidic acid (PA) [36,37]. Loss-of-function mutations in CTDNEP1 have been reported in medulloblastoma, a pediatric brain tumor [38]. Depletion of CTDNEP1 reduces diacylglycerol levels in the nuclear membrane and promotes NE rupture in cultured human cells [39,40]. However, the mechanistic link between diacylglycerol reduction and NE rupture, as well as its relevance to medulloblastoma pathogenesis, remains unknown. Additional evidence comes from studies of LBR, an inner nuclear membrane-localized protein containing a cholesterol reductase domain that is essential for cellular cholesterol synthesis [41]. LBR is up-regulated during fibroblast cell immortalization [42] and in metastatic melanoma [43]. In melanoma cells, LBR up-regulation promotes NE rupture in a manner dependent on its cholesterol reductase domain [43], suggesting an association between cholesterol increase and NE rupture. Whether this association reflects increased cholesterol content of the nuclear membrane remains unexplored.

### Mechanical stress: from outside and inside the cell

Mechanical forces applied to cells are well-described contributors to NE rupture (Figure 1B). For example, cell migration through constricted spaces of ∼2 × 5 μm^2^, a size smaller than the typical nucleus diameter (∼10 μm), induces NE rupture [44–46]. Similarly, experimental vertical compression of cells to a height of ∼3 μm induces NE rupture [18,19,47,48]. Interestingly, NE rupture does not occur directly at sites of compression or constriction. Instead, it often (but not always) occurs in small outward bulges of the NE, termed ‘nuclear blebs,’ that form due to cell compression or constriction [44–46,49–51]. Within nuclear blebs, Lamin B is depleted, likely because the high membrane curvature of blebs limits local assembly or retention of the Lamin B network. This local Lamin B loss renders blebs highly susceptible to rupture [52–55]. Supporting this model, reduction of nuclear lamins further increases the frequency of NE rupture under mechanical stress [9,18]. The duration between compression or constriction and NE rupture appears highly variable among cells (∼5 min to over 30 min) for reasons that remain poorly understood [21,40,44,45,50,55,56]. Emerging studies suggest that similar compression-driven NE rupture occurs in tissue contexts *in vivo* [43,44,57,58].

Forces generated by cytoskeletal movements within cells can also induce NE rupture. One example is the contraction of actomyosin fibers overlying the nucleus, which can induce NE rupture in various adherent cell types [50,56,59]. In contrast, perinuclear microtubule dynamics, rather than actomyosin contraction within sarcomeres, play a major role in promoting NE rupture in cardiomyocytes with Lamin A/C deficiency [14]. Mechanistically, both actomyosin- and microtubule-mediated forces are thought to exert compressive stress on the nucleus, creating conditions similar to those generated by external cell compression discussed above [14,50,56]. Shear stress may also contribute to NE rupture in skeletal muscles with Lamin A/C deficiency, as NE rupture occurs during microtubule-driven nuclear migration through the sarcomere network [15]. Because cytoskeletal organization differs substantially among cell types, the relative contributions of specific cytoskeletal networks to NE rupture are likely context dependent.

The physical connection between the nucleus and the cytoskeleton is mediated by the LINC complex at the NE (Figure 1C) [60–62]. The LINC complex consists of SUN domain proteins (SUN1 and SUN2) in the inner nuclear membrane and KASH domain proteins (Nesprins) in the outer nuclear membrane. KASH-domain proteins connect to actin filaments, microtubules, or intermediate filaments such as vimentin and desmin, thereby tethering distinct cytoskeletal networks to the NE [63]. The LINC complex plays a key role in transmitting cytoskeletal forces to the nucleus [64,65]. This normal force transmission contributes to NE rupture in the setting of Lamin A/C or Lamin B deficiency, as disruption of the LINC complex reduces NE rupture (Figure 1C) [8,15,50]. Interestingly, the LINC complex also mediates desmin-dependent outward forces on the NE, which counteracts microtubule-dependent inward forces and helps maintain nuclear shape in cardiomyocytes [66].

Non-cytoskeletal intracellular forces can also cause NE rupture (Figure 1D). One example is micron-sized cytoplasmic lipid droplets, which can indent the NE and cause NE rupture in cultured cells [67]. In this case, rupture occurs directly at sites of lipid droplet contacts [67], similar to NE rupture induced by experimental focal NE indentation [68], suggesting that inward high-curvature bending can cause NE rupture. Active transcription represents another source of non-cytoskeletal force. By generating substantial chromatin motion, transcription can contribute to NE rupture in both wild-type and Lamin B1-deficient fibroblasts [69,70].

Together, current evidence supports a model in which NE rupture reflects a mechanical imbalance between forces acting on the nucleus and the ability of the nucleus to resist those forces. Both increased mechanical stress and reduced nuclear resilience contribute to NE rupture susceptibility.

## Molecular consequences of NE rupture

An inevitable consequence of NE rupture is the loss of nucleo-cytoplasmic compartmentalization. We next discuss studies investigating the consequences of NE rupture (Figure 2).

**Figure 2:**
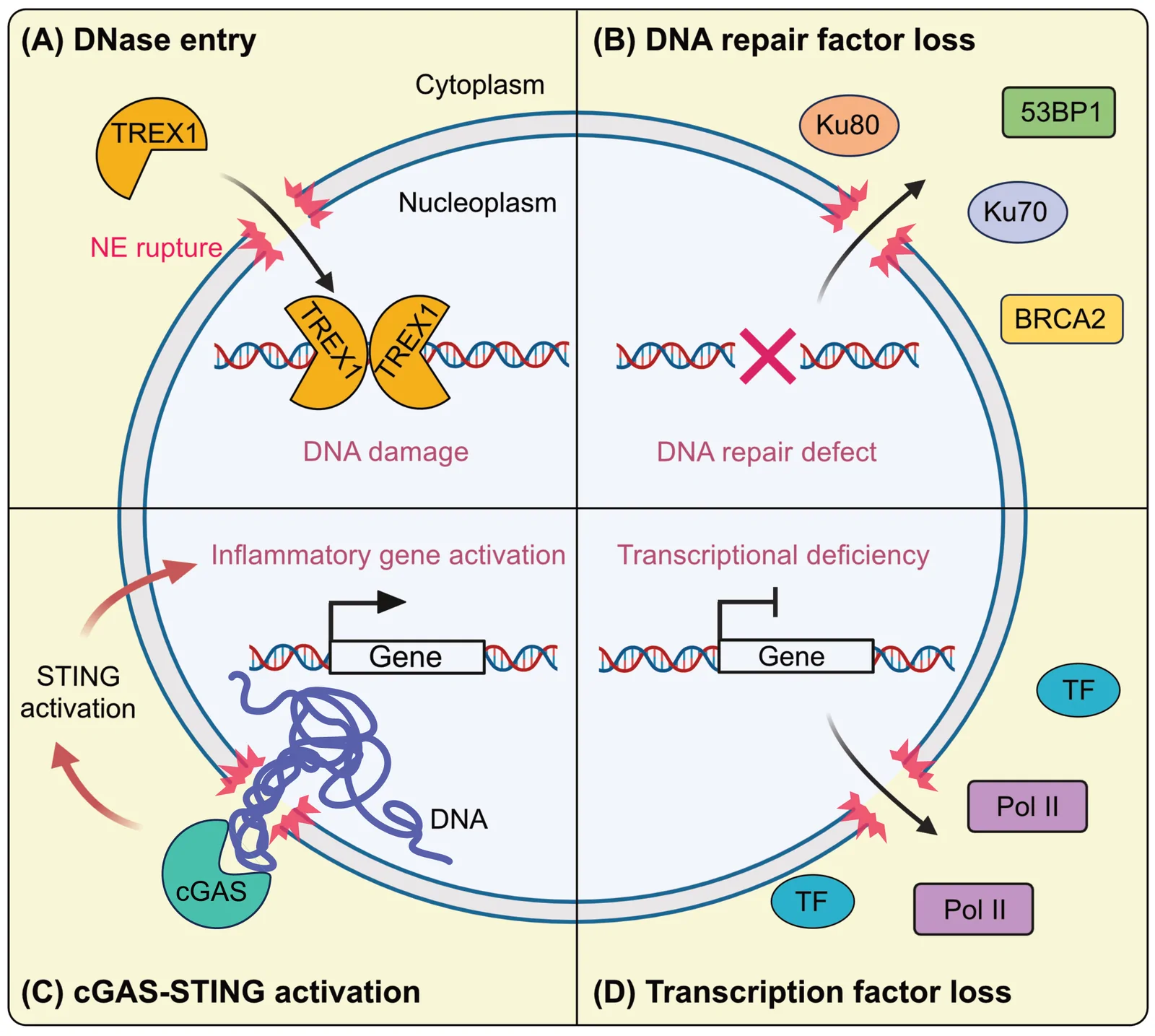
Consequences of NE rupture (**A**) NE rupture permits the entry of the cytoplasmic DNase TREX1 into the nucleus, leading to DNA damage. (**B**) NE rupture causes the leakage of DNA repair factors from the nucleus, compromising DNA repair. (**C**) NE rupture exposes nuclear DNA to the cytoplasm, activating the cGAS-STING cytosolic DNA-sensing pathway of the innate immune response. (**D**) NE rupture results in the leakage of RNA polymerase II and transcription factors (TFs), leading to global transcriptional deficiency.

### DNA damage: cause, consequence, or associated event?

A widely observed feature of NE-ruptured nuclei is increased DNA damage, as evidenced by the accumulation of DNA damage response markers γH2AX and 53BP1 [44,45]. This observation led to the hypothesis that NE rupture causes DNA damage. Supporting this idea, TREX1, an endoplasmic reticulum (ER)-localized DNA exonuclease, enters the nucleus upon rupture and contributes to DNA damage in cancer cell lines [58] (Figure 2A). Other studies suggest that DNA damage arises from the cytoplasmic mislocalization of DNA repair factors following NE rupture, rather than from TREX1 entry into the nucleus [9,68,71] (Figure 2B).

However, DNA damage does not appear to be a universal consequence of NE rupture. In Lamin A/C-deficient cardiomyocytes, NE rupture and DNA damage do not appear to be strongly correlated [13,72]. Furthermore, nuclear deformation itself can induce DNA damage independently of NE rupture [48,55]. The relative contributions of nuclear deformation and NE rupture to DNA damage appear to vary among cell types, potentially explaining some of the conflicting observations [48,55]. Conversely, several studies suggest that DNA damage can itself promote NE rupture through Lamin A/C phosphorylation [73] or heterochromatin softening [74,75] via activation of the DNA damage-responsive kinases ATR or ATM. These findings indicate that DNA damage may function both upstream and downstream of NE rupture.

### Cytosolic DNA sensing: elusive cGAS-STING activation

NE rupture may trigger innate immune responses through exposure of nuclear DNA to the cytoplasm. One prevailing hypothesis is that NE rupture activates the cGAS-STING cytosolic DNA sensing pathway of the innate immune response (Figure 2C). In this pathway, cGAS binds double-stranded DNA in the cytoplasm and produces the second messenger cGAMP, which activates the ER-localized adaptor STING. STING activation subsequently induces IRF3- and NFkB-dependent transcription of interferons and other innate immunity-related genes [76].

The hypothesis that NE rupture activates cGAS-STING originated largely from studies of micronuclei. Micronuclei contain missegregated chromosomes enclosed within their own NE, which is highly prone to rupture [77,78]. Following micronuclear rupture, cGAS accumulates on exposed chromatin and has been reported to promote interferon gene expression [79–81]. However, more recent studies have shown that cGAS association with micronuclei does not activate STING [82–84]. This may be because cGAS binds nucleosomal DNA exposed by micronuclear rupture, a binding mode that inhibits cGAMP production by cGAS [82–84].

Direct evidence that NE rupture of primary nuclei activates cGAS-STING is even more limited [17,49,85]. For example, in a mouse model of *LMNA*-associated dilated cardiomyopathy, neither cGAS accumulation at rupture sites nor STING-dependent interferon transcription was observed, owing to the very low endogenous expression of cGAS in cardiomyocytes [13,86]. This finding raises the possibility that cardiomyocytes may be generally incapable of mounting cGAS-STING responses to cytosolic DNA, including DNA released from damaged mitochondria. Together, current evidence suggests that cGAS expression levels and the chromatin state at rupture sites are important determinants of cGAS-STING activation following NE rupture. In addition, other cytosolic DNA sensors, including AIM2 [87,88], DNA-dependent protein kinase [89], and Ku70 [90], may also respond to rupture-exposed DNA to induce the innate immune response, although their roles in the context of NE rupture remain poorly understood.

### Leakage of nuclear factors: DNA repair and transcription factors

Given the loss of nucleo-cytoplasmic compartmentalization, leakage of nuclear proteins is another compelling hypothesis for the consequences of NE rupture. As discussed above, cytoplasmic mislocalization of several DNA repair proteins, including 53BP1, Ku80, Ku70, RPA2, and BRCA2, has been reported following NE rupture [68,71] (Figure 2B). Evidence suggests that loss of these factors from the nucleus impairs DNA repair and thereby contributes to DNA damage accumulation [9,53,68,71]. Notably, 53BP1 can remain mislocalized in the cytoplasm for several hours after rupture, potentially exerting prolonged effects on DNA repair fidelity [68].

By contrast, the leakage of transcription factors (TFs) and its biological consequences have received little attention. An early study reported cytoplasmic mislocalization of the TF OCT1 in Lamin A/C-deficient fibroblasts [20]. More recently, a direct relationship between NE rupture, loss of RNA polymerase II, and reduced transcriptional activity was demonstrated in Lamin A/C-deficient cardiomyocytes [72] (Figure 2D). Additional nuclear factors that are released from ruptured nuclei, and the functional consequences of their loss, remain largely unknown.

Collectively, these observations indicate that the consequences of NE rupture extend beyond the transient loss of compartmentalization. NE rupture can influence genome maintenance, innate immune signaling, and transcriptional regulation, thereby altering cellular function and state.

## Resealing of ruptured nuclear envelopes

Many studies have shown that NE rupture is transient and that nuclei experiencing NE rupture can eventually regain nuclear compartmentalization. These observations indicate that ruptured NEs can be resealed through endogenous mechanisms. We next discuss mechanisms that have been demonstrated or proposed to mediate NE resealing (Figure 3). Of note, we use the terms ‘sealing’ and ‘resealing’ to refer to re-establishment of nuclear compartmentalization, not necessarily restoration of continuous nuclear membranes at rupture sites.

**Figure 3:**
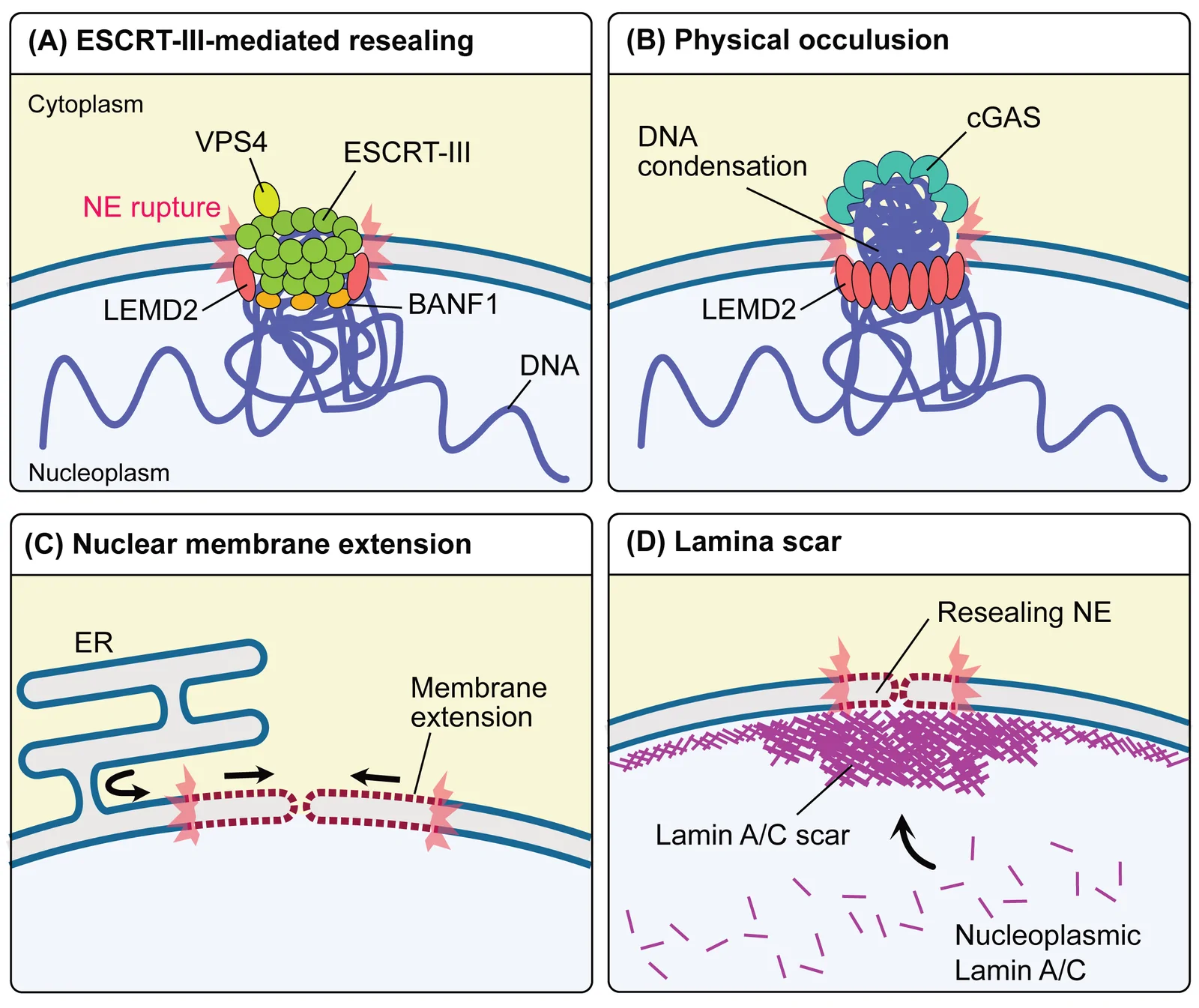
Mechanisms of NE rupture resealing (**A**) BANF1 and LEMD2 recruit the ESCRT-III complex and VPS4 to NE rupture sites, promoting nuclear membrane resealing. (**B**) BANF1-driven condensation of chromatin, cGAS, and LEMD2 at NE rupture sites may facilitate nuclear recompartmentalization through physical occlusion. (**C**) Lipid flow from adjacent nuclear membranes or the ER may promote nuclear membrane narrowing at rupture sites. (**D**) A nucleoplasmic pool of lamin A/C rapidly accumulates at rupture sites, forming ‘lamina scars’ that stabilize the resealing NE.

### The BANF1–ESCRT-III pathway

The BANF1–ESCRT-III pathway is the best-characterized mechanism of NE rupture sealing [21,44,45,91–96] (Figure 3A). The process closely resembles the mechanism by which this pathway mediates NE reassembly during mitosis [97]. First, a cytoplasmic pool of BANF1 (also known as barrier-to-autointegration factor, BAF) binds DNA exposed to the cytoplasm following NE rupture. BANF1 then recruits the inner nuclear membrane protein LEMD2, which promotes membrane recruitment to the surface of the exposed DNA. LEMD2 subsequently recruits the ESCRT-III adapter CHMP7, which in turn recruits the ESCRT-III membrane remodeling machinery. Finally, ESCRT-III remodels nuclear membranes to resolve membrane discontinuities with assistance from the AAA-ATPase VPS4 [44,45,91,94,97,98].

The kinetics of BANF1–ESCRT-III-mediated rupture sealing have been investigated in cultured human cells. BANF1 acts as a ‘first responder,’ accumulating at rupture sites within 15 s of rupture [91,98,99]. LEMD2 is recruited within approximately 100 s [99], followed by CHMP7 within 100–300 s [99]. Recruitment of the ESCRT-III components CHMP4B and VPS4 has been observed within 2 min of rupture [44,45]. However, the time required to fully restore nuclear compartmentalization varies markedly among cells, ranging from minutes to hours, even within the same cell type subjected to the same rupture insult, for reasons that remain poorly understood [20,21,23,25,40,45,94].

Accumulation of BANF1–ESCRT-III proteins at NE rupture sites has been observed across diverse types of rupture insults, in multiple cell types, in both proliferating and post-mitotic cells, and in both cultured cells and *in vivo* [44,45,72,91,94–96,100–102]. Together, these findings suggest that the BANF1–ESCRT-III pathway functions broadly across diverse pathophysiological contexts.

How is the ESCRT-III recruited specifically to NE rupture sites despite that both BANF1 and LEMD2 are constitutively present at the inner nuclear membrane? Local disruption of nucleocytoplasmic transport near NE rupture sites appears to serve as an important signal for site-specific ESCRT-III recruitment. In intact nuclei, CHMP7 is actively exported from the nucleus, preventing LEMD2-CHMP7 interaction at the inner nuclear membrane [103]. Following NE rupture, however, CHMP7 export is disrupted locally, permitting LEMD2-CHMP7 interaction at rupture sites [77,103]. In parallel, LRRC59 suppresses LEMD2-CHMP7 interaction in intact nuclei by binding LEMD2 and masking its CHMP7 binding site [104]. Upon NE rupture, defective importin-mediated release of LRRC59 causes its depletion near rupture sites, thereby enabling LEMD2 to engage CHMP7 [104].

### Other mechanisms of rupture sealing

The BANF1–ESCRT-III pathway is unlikely to be the sole mechanism responsible for NE rupture sealing, as inhibition of ESCRT-III recruitment or activity does not completely prevent restoration of nuclear compartmentalization [45,94]. One explanation is progression through mitosis, during which normal NE breakdown and reassembly reset NE architecture and resolve prior NE rupture [105–107].

Several additional mechanisms have been proposed to function during interphase. One mechanism involves chromatin compaction at NE rupture sites, which may serve as a permeability barrier [98] (Figure 3B). Such compaction is likely mediated by BANF1, whose DNA-binding activity can strongly condense chromatin [108,109]. Another possibility is physical occlusion of rupture sites by protein condensates. Both LEMD2 and cGAS contain intrinsically disordered regions capable of undergoing liquid-liquid phase separation [110–112], raising the possibility that condensates formed by these proteins contribute to temporary sealing of rupture sites. Recent work in fission yeast *Schizosaccharomyces japonicus* proposed an ESCRT-III-independent pathway, in which Alx1 (human ALIX) and Vid27 (no known human homolog) promote NE resealing during mitosis, although the underlying mechanism remains elusive [113,114].

Nuclear membrane lipid remodeling also contributes to NE rupture sealing, either together with or independently of ESCRT-III. Studies in yeasts have implicated PA, a lipid species that accumulates at highly curved nuclear membranes such as at NE rupture sites [115], in ESCRT-III recruitment. In *Saccharomyces cerevisiae*, PA-rich membranes directly interact with CHMP7 [115], whereas in *Schizosaccharomyces pombe*, PA binds the fission yeast*-*specific protein Bqt4, which in turn interacts with LEMD2 [116]. Because CHMP7 and LEMD2 are central mediators of ESCRT-III recruitment, these findings support a PA-driven pathway for directing ESCRT-III to NE rupture sites. In addition, increased flow of nuclear membrane lipids toward NE rupture sites may facilitate sealing of membrane gaps independently of ESCRT-III (Figure 3C). One candidate mediator is the very-long-chain fatty acid elongase Elo2 in *S. pombe* (human orthologs ELOVL1–7), whose overexpression reduces NE rupture in the absence of ESCRT-III recruiters [117]. The contribution of membrane flow to NE resealing is likely influenced by rupture size, which can range from 50 to 500 nm depending on the nature of the rupture-inducing insult [13,25,45,52,118,119].

### The nuclear envelope after resealing

Whether the NE fully returns to its pre-rupture state following resealing remains an open question. Lamin A/C is re-recruited to rupture sites from its nucleoplasmic pool through interaction with BANF1 and forms thick patch-like assemblies often referred to as ‘scars’ [45,94,96] (Figure 3D). Lamin A/C scars arise within minutes of laser-induced rupture in fibroblasts [96] and persist for hours following migration-induced rupture in cancer cells [45]. These structures are thought to prevent recurrent NE rupture at the same site [120], a phenomenon frequently observed in cells deficient for Lamin A/C or Lamin B1 [20,21,94]. By contrast, Lamin B1 and nuclear pores do not appear to be efficiently restored at resealed sites [45,55,121]. In proliferating cells, the abnormal state of resealed NEs generated during interphase is likely reset during subsequent mitotic NE breakdown and reassembly. In post-mitotic terminally differentiated cells, such as cardiomyocytes and neurons, however, the long-term consequences of persistent alterations in resealed NEs may be more significant.

Collectively, current evidence suggests the BANF1–ESCRT-III pathway as the principal mechanism of NE rupture sealing. Multiple chromatin- and lipid-derived signals generated by rupture ensure timely and regulated ESCRT-III recruitment, while additional mechanisms may provide complementary routes for restoring nuclear compartmentalization.

## Nuclear envelope rupture in physiology and pathology

NE rupture has been observed across a wide range of cell types and disease contexts *in vivo* [9,13–15,17,18,24,31,43–45,57,58,68,72,86,122,123]. While the association between NE rupture and disease has historically been correlative, emerging studies support a more direct role for NE rupture in disease pathogenesis.

### Laminopathy

One of the strongest lines of evidence linking NE rupture to disease comes from studies of *LMNA-*related dilated cardiomyopathy (LMNA-DCM), a form of laminopathy (Figure 1A). LMNA-DCM develops ventricular chamber dilation, cardiac conduction defects, and progressive loss of contractile function, ultimately leading to cardiac arrest or heart failure [124,125]. NE rupture has been observed in hearts from both LMNA-DCM patients and mouse models [8,10,11,13,14,126]. Inhibition of the ESCRT-III-mediated NE rupture sealing in mouse models exacerbates cardiomyopathy, suggesting that NE rupture contributes to disease progression, whereas ESCRT-III-mediated resealing is protective [72]. Consistent with this idea, disruption of the LINC complex prevents NE rupture and ameliorates cardiac dysfunction in multiple mouse models [8,14,86,127] (Figure 1C). NE rupture in LMNA-DCM mice is associated with loss of RNA polymerase II and transcriptional deficiency, which may explain the widespread down-regulation of genes critical for cardiomyocyte function [72]. Thus, NE rupture-induced transcriptional deficiency emerges as a potential pathogenic mechanism in LMNA-DCM.

NE rupture has also been reported in a mouse model of Hutchinson–Gildford progeria syndrome (HGPS), another laminopathy caused by a splice-site mutation in *LMNA* [128]. A critical pathological feature of HGPS is the loss of aortic SMCs, which promotes development of life-threatening arteriosclerosis [129]. In a mouse model of HGPS, NE rupture occurs in aortic SMCs but not cardiomyocytes and is associated with increased DNA damage and SMC loss [17]. Together, studies of LMNA-DCM and HGPS support NE rupture’s contribution to laminopathy pathogenesis. Whether NE rupture similarly contributes to other laminopathies, such as Lamin A/C-related lipodystrophy or Lamin B1-associated leukodystrophy, remains unclear.

### Cancer

Multiple studies have documented NE rupture in cancer [43,58,100] (Figure 1B). In glioblastoma, NE rupture is particularly prominent at invasive tumor margins and in cells with reduced Lamin A/C expression [58,100]. Because reduced Lamin A/C expression increases nuclear deformability and facilitates cell migration through confined environments [130–132], cancer cells may gain migratory capacity at the expense of increased susceptibility to NE rupture [100]. NE rupture itself may further facilitate migration by transiently relieving mechanical constraints imposed by the nucleus. Interestingly, DNA damage associated with NE rupture has been proposed to promote extracellular collagen degradation through currently unknown mechanisms, potentially creating a microenvironment favorable for invasion and metastasis [58].

Because NE rupture is frequently associated with DNA damage, one attractive hypothesis is that it contributes to genomic instability during tumor progression. Supporting this possibility, severe NE rupture can generate cytoplasmic chromatin fragments resembling micronuclei in migrating cancer cells [45]. However, mechanical compression independently induces abnormal mitosis, leading to DNA missegregation, micronucleus formation, and genomic instability in cancer cells without requiring NE rupture [133]. Thus, while current evidence supports a role for NE rupture in promoting cancer cell invasiveness, its contribution to genomic instability remains unresolved.

### Neurodegenerative disease

Neurodegenerative diseases have recently emerged as potential NE rupture-associated disorders [101,134,135] (Figure 1D). These diseases are often characterized by aggregation of tau, alpha-synuclein, or polyglutamine proteins within the nucleus or cytoplasm [136]. Such protein aggregates have been associated with NE rupture or deformation, raising the possibility that protein aggregates physically damage the NE [101,135,137–139]. Aging may further increase vulnerability to these insults, as Lamin B1 expression declines in neural cells in the brain of both aged mice and humans [140–142].

Interestingly, a recent study suggests that the relationship between protein aggregation and NE rupture may be bidirectional. A genetic screen for suppressors of Tau aggregation identified several proteins involved in NE rupture sealing, including BANF1, LEMD2, ANKLE2, and CHMP7 [143]. BANF1 and ANKLE2 depletion increases Tau phosphorylation at Ser 356 in neurons, a modification that weakens Tau-microtubule interactions and may facilitate aggregation [143]. Thus, a feed-forward cycle may exist between NE rupture and protein aggregation in neurodegenerative disorders.

### Nuclear envelope rupture in physiology

NE rupture also occurs during physiological processes. In *Drosophila* wings, leukocytes exhibit NE rupture while migrating through narrow vascular-like channels during immune surveillance [57] (Figure 1B). However, the frequency of NE rupture is minimized by adaptive reduction of Lamin C, which facilitates nuclear deformation and thereby protects cells from excessive mechanical stress [57]. In humans, cells within the basal layer of stratified cervical epithelium exhibit frequent NE rupture, potentially due to low Lamin B1 expression, and this phenomenon has been proposed to facilitate papillomavirus infection [102].

An example of NE rupture with a well-established physiological function occurs during neutrophil extracellular trap formation [144]. During this process, chromatin decompaction and Lamin B disassembly drive regulated NE rupture, allowing release of extracellular DNA that facilitates pathogen capture and elimination [145,146]. Given that lamin expression changes dynamically during development, differentiation, and aging [33–35,140–142,147,148], additional physiological settings likely exist in which NE rupture occurs in response to endogenous mechanical stress.

Collectively, these findings broaden the biological significance of NE rupture beyond laminopathies to encompass diverse physiological and pathological settings, including immune surveillance, host defense, cancer progression, and neurodegeneration. Understanding how the causes, consequences, and cellular responses to NE rupture differ among these contexts remains a major challenge for the field.

## Summary and outlook

The last decade has witnessed major advances in our understanding of the biochemical and biophysical mechanisms underlying NE rupture and resealing. Observations of NE rupture in diverse physiological and pathological contexts *in vivo*, together with growing insight into its consequences, have expanded the biological significance of this phenomenon. Despite this progress, several important knowledge gaps exist:

### Physiological and pathological contexts in which NE rupture occurs

NE rupture likely occurs in many more biological settings than are currently recognized. Improved tools for detecting NE rupture and resealing, such as combinations of fluorescent nuclear-localized reporters and catalytically inactive cGAS-based sensors [72], should facilitate the identification of additional physiological and pathological contexts in which NE rupture occurs.

### Short-term and long-term consequences of NE rupture

A systematic understanding of the molecules and organelles that enter or exit the nucleus during NE rupture remains lacking. Defining the immediate and long-term consequences of NE rupture, including DNA damage accumulation, chromatin alterations, and transcriptional dysregulation, will be essential for understanding its pathophysiological significance.

### Molecular and biophysical basis of NE rupture and resealing

Many fundamental aspects of NE rupture and resealing remain poorly understood. These include the biophysical principles governing rupture initiation and repair, the kinetics of these processes, the lipid composition and organization of ruptured membranes, the chromatin and transcriptional states of ruptured and resealed nuclei, the genomic regions exposed at rupture sites, and the full repertoire of proteins recruited during NE resealing.

### Genetic and pharmacologic approaches to manipulate NE rupture and resealing

Potential intervention points include extracellular mechanical forces, intracellular mechanotransduction pathways, NE stability, and NE resealing mechanisms. Development of approaches to manipulate these processes may create therapeutic opportunities for NE rupture-associated conditions, including laminopathies, neurodegenerative diseases, and cancer.

In summary, NE rupture has emerged as a fundamental cellular process with the potential to explain diverse disease mechanisms. Addressing these outstanding knowledge gaps will advance our understanding of the roles of NE rupture and resealing in development, tissue homeostasis, aging, and disease.

## Perspectives

- Importance of the field: NE rupture has been observed in a broad range of physiological and pathological contexts, including development, cancer, and degenerative diseases. However, its contribution to tissue homeostasis and disease pathogenesis remains unclear.
- Current thinking: NE rupture arises from an imbalance between mechanical forces acting on the nucleus and the ability of the NE to resist those forces. It has been associated with DNA damage, transcriptional dysregulation, and cytosolic DNA-mediated immune responses, although the mechanisms and generality of these processes remain controversial. NE rupture is often transient because endogenous NE resealing mechanisms restore nuclear compartmentalization. The BANF1-ESCRT-III pathway is the principal resealing mechanism identified to date, although alternative and complementary pathways may also contribute to NE resealing.
- Future directions: Identifying additional physiological and pathological contexts in which NE rupture occurs will improve our understanding of its biological role. Defining both immediate and long-term consequences of NE rupture will be critical for understanding its pathophysiological impact. Elucidating the molecular and biophysical mechanisms governing NE rupture and resealing will provide insight into how these processes influence cellular functions. Finally, the development of genetic, pharmacological, and biophysical modeling approaches to manipulate NE rupture and resealing may open new therapeutic avenues for disorders associated with NE rupture, including laminopathies, neurodegenerative diseases, and cancer.

## Open Access

Open access for this article is provided via our Subscribe to Open programme, made possible through the support of our subscribing institutions. Learn more here: https://portlandpress.com/pages/open_access_options_and_prices#conditional.

## Abbreviations

ER: endoplasmic reticulum
HGPS: Hutchinson–Gildford progeria syndrome
LINC: linker of nucleoskeleton and cytoskeleton
LMNA-DCM: LMNA-related dilated cardiomyopathy
NE: nuclear envelope
PA: phosphatidic acid
SMCs: smooth muscle cells
TFs: transcription factors
